# Colour formation on the wings of the butterfly *Hypolimnas salmacis* by scale stacking

**DOI:** 10.1038/srep36204

**Published:** 2016-11-02

**Authors:** Radwanul Hasan Siddique, Silvia Vignolini, Carolin Bartels, Irene Wacker, Hendrik Hölscher

**Affiliations:** 1 Institute of Microstructure Technology, Karlsruhe Institute of Technology (KIT), Hermann-von-Helmholtz-Platz 1, 76344 Eggenstein-Leopoldshafen, Germany; 2 Department of Chemistry, University of Cambridge, Lensfield Road, Cambridge CB2 1EW, UK; 3 Centre for Advanced Materials (CAM), University of Heidelberg, 69120 Heidelberg, Germany

## Abstract

The butterfly genus *Hypolimnas* features iridescent blue colouration in some areas of its dorsal wings. Here, we analyse the mechanisms responsible for such colouration on the dorsal wings of *Hypolimnas salmacis* and experimentally demonstrate that the lower thin lamina in the white cover scales causes the blue iridescence. This outcome contradicts other studies reporting that the radiant blue in *Hypolimnas* butterflies is caused by complex ridge-lamellar architectures in the upper lamina of the cover scales. Our comprehensive optical study supported by numerical calculation however shows that scale stacking primarily induces the observed colour appearance of *Hypolimnas salmacis*.

Many butterflies (Order: Lepidoptera) possess very fascinating colouration, and most species of butterfly can be identified solely by the colour pattern on their wings^1^,^2^,^3^. Such a diversity has attracted a wealth of research to determine the mechanisms responsible for such colours^4^,^5^,^6^. From a functional perspective, wing colouration can be important in a multitude of ways, ranging from mating^3^,^7^, camouflage^8^,^9^ and warning purposes^10^,^11^. Moreover, structurally unique, visually chromatic and complex colour mechanisms found in numerous butterfly wings inspired various advanced technical applications. For example, a hierarchical multilayer air-cuticle pattern inspired by *Morpho* butterflies was mimicked for selective gas sensing^12^ and colourful hydrophobic coatings^13^. Colour mixing due to multilayer microcavities inspired from *Papilio blumei* was replicated for polarization-sensitive optical signatures^14^. The reverse diffracting grating effect of *Pierella luna* was copied artificially and might be useful for bio-sensing and anti-counterfeiting^15^. Hence, analysing butterfly colour patterns does not only enrich our understanding of inter/intra-specific communication and the evolution of exaggerated signalling of butterflies, it can also be the source of new photonic devices.

*Hypolimnas salmacis*, also known as the “Blue Diadem”, is a butterfly in the family *Nymphalidae* from the Afro-tropic eco-zone^16^ (Fig. 1a). It is one of 23 butterflies found in the genus *Hypolimnas*^16^ that is well-known for its sexual dimorphism. In other words, the two sexes of the same species are distinct in colour, shape, size, and structure. On the dorsal wings, almost all *Hypolimnas* species, including *Hypolimnas salmacis*, carry UV-white, blue and brown/black patches^17^. As the respective colours, including their brightness and saturation, are crucial for the animals’ selective mate, species or rival recognition^18^,^19^, it is of utmost importance to understand the basis of their colouration. Studies on *Hypolimnas bolina* suggested that structurally coloured ornaments serve as sexual signals^20^ and that they carry potentially useful information on phenotypic quality^21^. After investigating the reflectance of these butterfly wings, Kemp *et al*.^22^ reported that the angle-dependent reflectance arises as a consequence of the optical mechanisms including constructive interference and diffraction at the complex ridge-lamellar formation of the upper lamina. In a more recent study on another genus, *Hypolimnas alimena*, the dull colouration mechanism of the scales is explained via simpler micro-architecture of the upper lamina as well^19^. Here, we analyse the colour formation on the wings of *Hypolimnas salmacis* and show that its blue iridescence is rather caused by the lower thin lamina of the cover scale. We demonstrate explicitly that the blue colouration arises primarily from the stacking of brown and white scales on the wing.

## Results

### Optical appearance of scales on the wing

**Figure 1:**
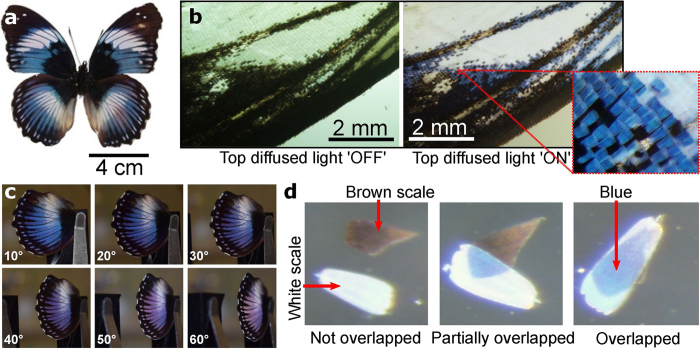
Scales of the butterfly *Hypolimnas salmacis*. (**a**) Photo of a *Hypolimnas salmacis* butterfly. Its dorsal wings feature three regions: blue, white and dark brown/black. (**b**) Optical image of the forewing showing all three regions without and with diffused light from the top. Blue coloured scales only appear under diffused light condition. A zoom shows the scales stacking on the wing. (**c**) By tilting of the wing, the colour of blue regions shifts to purple-violet. This effect demonstrates the iridescence of *Hypolimnas salmacis* butterfly wings. (**d**) Overlapping of single white and brown scales causes the blue appearance of the white scales and reveals the colouration mechanism by scale stacking.

The dorsal side of the *Hypolimnas salmacis* wings features dark brown or black, white, and blue regions. On the ventral side, white regions mimic the dorsal side while everywhere else the wings are covered by light brown scales. The basal region is almost black near the thorax for both forewing and hindwing. The outer margin of the whole wing is mostly brown and covered with blue and white patches in the middle. A stereo-microscopy image of the *Hypolimnas salmacis* wing is shown in Fig. 1b. Interestingly, the blue colouration is visible only in diffused illumination, suggesting that the origin of colouration is purely physical. Moreover, tilting the wing shifts the blue colour to purple-violet (Fig. 1c) that demonstrates the iridescent property of the *Hypolimnas salmacis* wing. As in most other *Lepidoptera* species, both white and black/brown areas are uniformly covered with cover and ground scales^4^.

As clearly visible in Fig. 1b, some of the cover scales in the blue areas are not completely blue. While examining thoroughly scales in blue areas, we observed that white cover scales stacked on brown ground scales appear blue. And, the same cover scales look white when lying on top of white ground scales. To further confirm our observation, we picked a single white cover and a single brown ground scale from the blue region. We manually overlapped the white cover and brown ground scales whereupon the blue colouration appears as a result of the stacking (see Fig. 1d). The cover scale appeared white when the bottom brown scale was absent. Hence, the formation of the blue colour on the *Hypolimnas salmacis* wings occurs due to scale stacking when a white scale is on top of a brown one.

### Electron microscopy of scales

**Figure 2:**
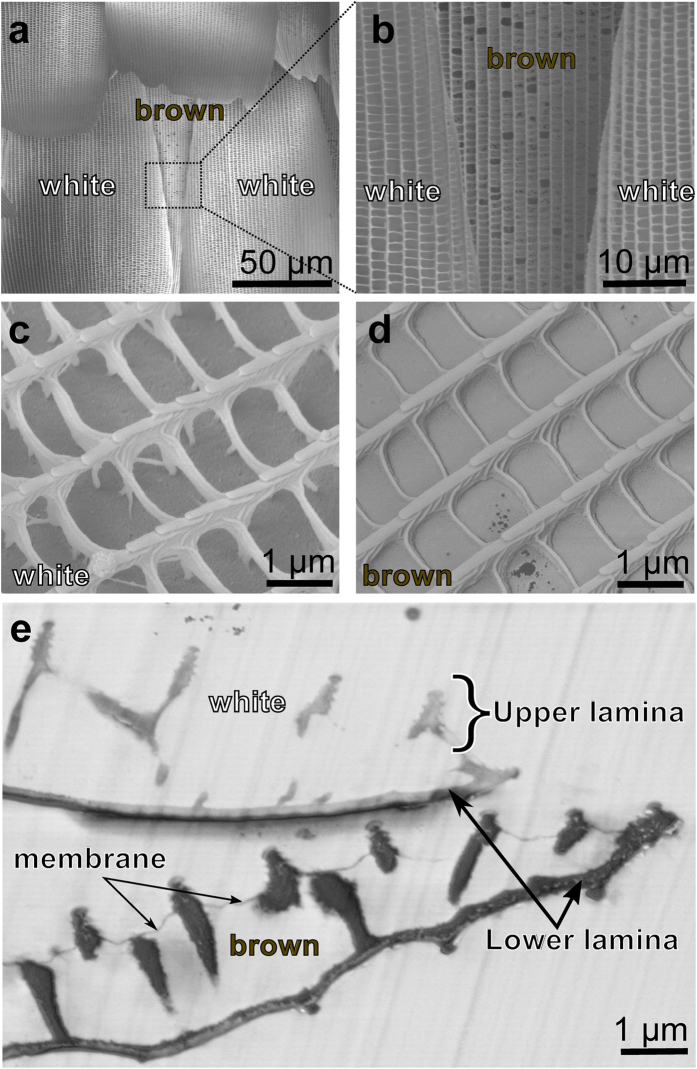
Micro- and nanostructure of the *Hypolimnas salmacis* scales. (**a**,**b**) SEM images of stacks of white cover scales on brown ground scales. (**c**) Detail of a single white scale. Longitudinal grating like ridges along the scale are connected with cross-ribs. The sides of the ridges are covered with small microribs. (**d**) Detail of single brown scale. In terms of dimension, it mimics almost exactly the white scale but the windows, created by the cross-ribs, are closed by thin pigmented membranes. (**e**) SEM image of a cross section of the forewing including a white and brown scale. Both scales show similar features with upper lamina of ridges and tiny microribs as well as lower lamina of thin films. Thin membranes between the ridges are visible only in the brown scale.

Creating blue colour out of white and brown does not accord with conventional colour blending or filtering mechanism. To understand this effect, we first analysed the scales by electron microscopy (Fig. 2a–d). The blue areas consist of stacks of white and brown scales (Fig. 2a,b) as already observed from the optical analysis. The SEM images of the white and brown scales show the typical oval shape with a width of 100 *μ*m and length of 200 *μ*m. Both of them consist of grating-like ridges with a typical distance of (2 ± 0.2) *μ*m. The brown scales feature thin membranes between the ridges (Fig. 2d) while the white ones do not (Fig. 2c). Cross-ribs across the ridges create open areas with a size of (2 ± 0.2) *μ*m × (1 ± 0.1) *μ*m. Such windows created by cross-ribs are commonly termed *alveoli* and found in *Papilionids*^6^. The sides of the ridges are covered with microribs. The cross-sectional SEM image of the blue region shown in Fig. 2e reveals longitudinal ridges of cover and ground scales both consisting of very small microribs. The ridges are standing on a single thin film (lower lamina) with a thickness of around (190 ± 20) nm. Thin membranes can be observed in the windows of the brown scales between the ridges (Fig. 2d,e).

### Optical spectroscopy of scales on the wing

**Figure 3:**
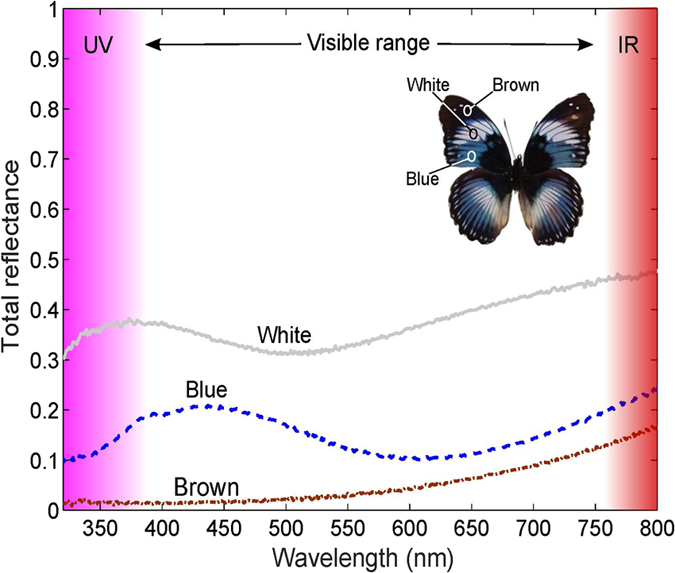
Reflectance spectra of *Hypolimnas salmacis* recorded on different wing areas. Total reflectance spectra of blue, white and brown regions measured as indicated in the inset. Blue areas show a considerably low intense reflection with a broad peak at ≈430 nm. No distinct peak is observed in the visible regime (380–760 nm) of the reflection spectra of the white area. In the UV, however, there is a broad peak at ≈375 nm. Brown scales barely reflect in the UV and the visible regime but the reflection increases towards the infrared.

Total diffusive reflection spectra were measured in different regions of the *Hypolimnas salmacis* wings with an integrating sphere. Figure 3 shows the obtained spectra of blue, white and brown regions marked in the inset. Blue areas feature a weak reflection of only 20% reflectance with a broad peak at ≈430 nm. In the reflection spectrum of the white area, there is no particular peak in the visible regime, resulting in the overall white appearance. Nonetheless, a considerably broad reflection peak can be noticed in the UV at ≈375 nm. A similar UV reflection was reported in the white patch of *H. bolina*^22^. Although the overall structure of the brown scales of *H. salmacis* is comparable to that of the white scales, the brown area has no reflection peak neither in UV nor in the visible spectral regime. This is presumably due to high melanin pigmentation in the membranes and ridges of the brown scales leading to high absorption.

### Optical spectroscopy of single scales

**Figure 4:**
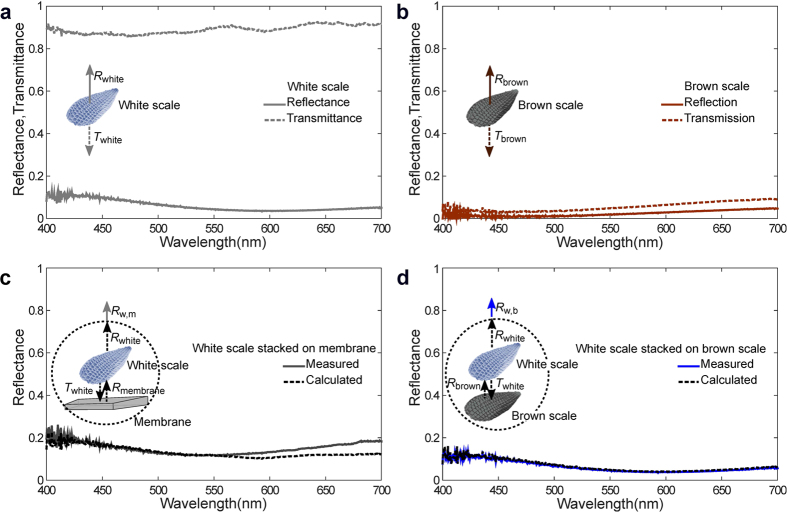
Single scale spectroscopy of *Hypolimnas salmacis*. (**a**) Spectra of a single white scale in reflection (solid line) and transmission (dashed line). (**b**) Spectra of a single brown scale in reflection (solid line) and transmission (dashed line). (**c**) Reflection spectrum measured on stack of a white scale on a wing membrane (solid line). Individually measured reflectance and transmittance properties are used to calculate the white scale-wing membrane stack reflection (dashed line) and compared with the experimental stack measurement (solid line). A schematics of a single white scale spectrometry on a wing membrane with individual reflectance and transmittance terms is provided in the inset. (**d**) Reflection spectrum measured on stack of a white and brown scale (solid line) which demonstrates the blue colouration due to the peak at the 420 nm. Individually measured reflectance and transmittance properties are also used to calculate (dashed line) and confirm the experimental stack measurement (solid line). The insets show a schematic of the measurement.

In order to understand the observed scale stacking effects in more details, we performed also micro-spectrometry on individual scales and stacks of scales. All the reflection measurements are performed on black background to avoid stray reflection/scattering. The resulting spectra are plotted in Fig. 4. The white scale is almost transparent in the visible regime with a high transmittance of ≈90% (dashed line, Fig. 4a. Thus, the white scale can be considered as nearly pigment-free. However, an isolated white scale has a reflection peak at 420 nm, i.e., in the blue spectral area (solid line, Fig. 4a). We carried out similar experiments on a single isolated brown scale (Fig. 4b). The resultant spectra indicate very low reflecting and transmitting properties of the brown scale and therefore point out its strong absorbing behaviour.

To explain how the appearance of white and blue is created by scale stacking, we first measured the reflection spectrum of white scales lying on the wing membrane of *Hypolimnas salmacis* (Fig. 4c). In this arrangement, the white appearance indeed coincides with a broad spectrum (grey line) over all visible wavelengths. The spectrum of the wing membrane itself shows also a broadband reflection and whitish colouration (not shown). Defining the individual reflectance of a white scale, a brown scale and a wing membrane as *R*_white_, *R*_brown_ and *R*_membrane_, respectively and the transmittance of white scale as *T*_white_, the reflectance of a white scale stacking on the wing membrane can be calculated from


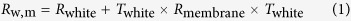


assuming non-coherent scattering during the light propagation in the stack. The resulting spectrum (black dashed line) is shown in Fig. 4c with a concise schematic noting the terms as inset. The calculated spectrum fits well with the measured stacked spectrum. However, some small mismatch can be noticed at long wavelengths, which might be due to a partial coherence effect during the light propagation which is not considered in the equation above^23^.

In a next step we placed a white scale on top of a brown scale and the measured reflection spectrum (solid blue line, Fig. 4d) shows a reflection peak at 420 nm in the same blue spectral area as observed in the reflection spectrum of the isolated white scale. These observations suggest that a white scale on an absorbing background exhibits blue colouration. To check the consistency of our measurements and assumptions we also calculated the non-coherent scattering of a white scale on a brown scale


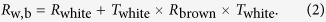


The calculated spectrum (black dashed line) of the stacked system in this case matches the measured spectrum completely as depicted in Fig. 4d. Hence, the overall result explains the scale stacking phenomena satisfactorily.

### Translucency of white scales

**Figure 5:**
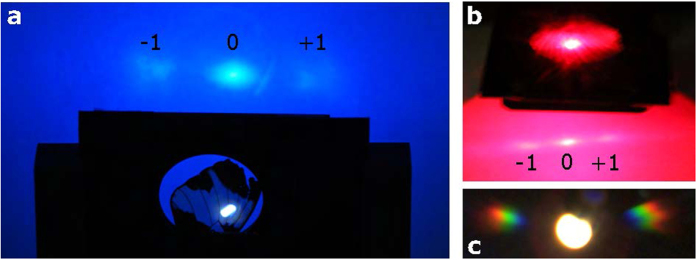
Light diffraction of the white scales of *Hypolimnas salmacis*. (**a**) A blue (445 nm) and (**b**) a red laser (635 nm) shine through the white region of a *Hypolimnas salmacis* wing. The resultant higher diffraction orders on the screen demonstrate the transmission grating like behaviour of the white scales. (**c**) K-space imaging of a single white scale in transmission mode showing high order diffraction.

The above presented micro-spectroscopy of a white scale resulted in a transmittance of about 90%. However, this experiment does not provide the scattering properties, i.e. whether white scales are transparent or translucent. In order to examine this property, we passed laser light with two different wavelengths (blue with 445 nm and red with 635 nm) through the white region of a butterfly wing and captured the resultant diffraction patterns on a screen (Fig. 5). A transparent medium does not allow diffuse light scattering and diffracts only the zeroth order. In our case, however, two diffracted orders including the zeroth order are observed on the screen indicating large diffuse light scattering at the micro-ridge patterns (2 ± 0.1 *μ*m) of the white scales^24^. The diffraction pattern of a single white scale shown in Fig. 5c was obtained in transmission mode using a K-space imaging system with Bertrand lens^25^. This conoscopic imaging allows to record the directionality of the scattered beam, i.e. to record the Fourier plane. The zeroth order corresponds to a direct transmission of the white light source through the scale. For higher orders, colours with increasing wavelengths (from blue to red) are diffracted at increasing angles. However, the higher order diffraction signals are relatively broader in angle due to the inherent disorder of the natural grating. This outcome clarifies that white scales act as a translucent medium in the visible regime. Due to the grating like ridge architecture, it scatters all wavelengths non-coherently if it lies on top of a white or random surface which diffuses the light.

### Simulation of the thin film interference of the white scales

The experimental results presented above already demonstrated that the effect of the blue colouration is caused by scale stacking which amplifies the broad blue peak of the white scales. As the upper lamina works as a diffuser, we speculate that the lower feature-less thin lamina is the origin of this blue peak. To prove that, we simulated the reflection of a thin film with a typical refractive index of chitin, i.e. *n*_c_ = 1.56^26^. The reflectance of a thin film surrounded by air at any incident angle of *θ* for a given polarisation can be calculated from^27^


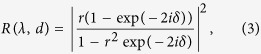


where *δ* = (2*πn*_c_*d* cos *θ*)/*λ* is the phase delay introduced by the film thickness of *d*, and *r* is the reflection coefficient at the air-chitin boundary governed by Fresnel’s equation for a given polarisation, i.e., *r* = (cos *θ* − *n*_c_ cos(sin^−1^(sin *θ*/*n*_c_)))/(cos *θ* + *n*_c_ cos (sin^−1^(sin *θ*/*n*_c_))) for s-polarisation or *r* = (cos(sin^−1^(sin *θ*/*n*_c_)) − *n*_c_ cos *θ*)/(cos(sin^−1^(sin *θ*/*n*_c_)) + *n*_c_ cos *θ*) for p-polarisation. Additionally, we have to consider that the thickness of the lamina is not perfectly constant. We observed in the electron microscopy images that it varies locally from 170 to 210 nm within the scale. In order to include these local variations of the thin film, we modeled the film thickness by a Gaussian distribution


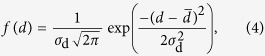


with mean thickness 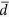 and variance *σ*_*d*_. In this way, the local thickness variation of the lamina can be easily combined with Eq. (3) and the averaged reflectance of the wavy membrane for a given polarisation can be calculated from


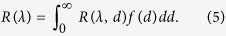


**Figure 6:**
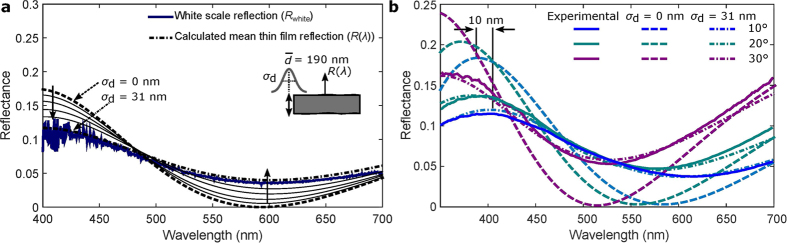
Simulation of the lower thin lamina of *Hypolimnas salmacis* white scales. (**a**) Thin film reflection is calculated at normal incidence considering a thin chitin film surrounded by air. In order to account for the local variations observed in the lower lamina of the white scales, the thickness of the film is modeled with a Gaussian distribution as shown in the inset with a mean thickness 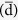 of 190 nm and a variance (*σ*_d_) of 31 nm. The simulated mean reflectance (dash-dotted line) of the distribution shows good agreement with the experimental reflection spectrum (solid line). (**b**) The experimental specular reflection spectra (solid line) at oblique incident angles are compared with the developed model in unpolarised light condition (average of s- and p-polarisation) for a mean thickness of 190 nm and a variance of 31 nm. Calculated reflection spectra of the bio-inspired thin film (dash-dotted line, *σ*_d_ = 31 nm) encounters a red-shift of 10 nm in the peak wavelength of reflection at oblique incident angles with respect to a simple flat thin film (dashed line, *σ*_d_ = 0 nm).

With this equation, first we calculated the thin film reflection for a mean thickness of 190 nm and varied the variance *σ*_d_ at normal angle of incidence (Fig. 6a). For *σ*_*d*_ = 0 nm, the calculated spectrum is simply the thin film reflectance for a 190 nm slab surrounded by air (dashed line). The effect of local variation on the reflection properties by the roughness factor *σ*_*d*_ is indicated by dotted arrows. The corresponding reflection spectrum is shown in Fig. 6a and compared with the single white scale reflectance obtained from the micro-spectrometry. The simulated thin film reflectance with a variance *σ*_*d*_ = 31 nm agrees well with the experimental data. Furthermore, we calculated the reflection spectra in unpolarised light condition by averaging out the s- and p-polarisation for various oblique angles of incidence and compared with the experimental ones in Fig. 6b. The roughness factor of *σ*_*d*_ = 31 nm in the model also corresponds well with the experimental data at off-normal light condition. A blue-shift is noticed in the peak wavelength of reflection spectra towards UV spectral region at large angles of incidence. However, the inclusion of roughness factor of *σ*_*d*_ = 31 nm in the thin film model red-shifts the peak wavelength of reflection of a flat thin film (*σ*_*d*_ = 0 nm) by 10 nm (Fig. 6b). Overall, the theoretical thin film model verifies that the blue colouration results from the thin film nature of the lower lamina.

## Discussion

Very little research on the butterfly *Hypolimnas salmacis* has been published so far although some other *Hypolimnas* species were studied extensively^19^,^22^,^28^. We observed structural UV reflection from the white patches owing to the multilayer microrib architecture. UV reflection from ridge-microrib structures has been already observed in *nymphalids, pierids* and other butterfly families^29^,^30^. However, to create blue reflection from a similar structure, longitudinal and transverse dimensions of the microribs need to be sufficiently large. This statement is supported by the *Morpho* butterfly structural pattern as a very good natural example of such architecture, as well as by several experimental biomimetic structures^12^,^31^,^32^. It was also reported that the origin of the intense and directional blue reflection of *Hypolimnas bolina* is similar kind of complex structures^22^.

However, we demonstrated that the colouration of the blue areas of the *Hypolimnas salmacis* wing is caused by scale stacking where the origin of the blue colour is the thin lower lamina of the white scales. Our experimental analysis as well as theoretical analytical modelling confirm that the phase delay introduced by the thin film lower lamina causes light interference in blue spectrum. Indeed, the lower lamina works as a one dimensional photonic crystal which is commonly found in butterfly wing scales^33^,^34^. However, due to the low index contrast between chitin (*n* = 1.56) and air (*n* = 1), the thin lamina can not form a complete photonic bandgap. It rather creates a pseudo-bandgap which causes the iridescence of the *Hypolimnas salmacis*^35^. The pseudo-photonic bandgap in the visible spectrum created by photonic crystals is responsible for iridescence in many other butterflies and insects^5^,^34^. Hence, the proposition of thin film interference explains the violet-purple appearance of the wing at oblique angles as shown in Fig. 1c. The theoretical modelling of a thin film including surface variation also confirms this hypothesis (Fig. 6b).

The local variation of the laminar thickness can be modelled by a Gaussian distribution and the variance (standard deviation) can act as a roughness factor. Such surface roughness smoothens the reflection spectrum of a single thin flat film (Fig. 6a). This explains the dull appearance of the blue colour. Although an isolated white scale has a peak blue reflectance of around 10%, blue areas on the wing reach up to 20% due to multiple scattering of overlapping scales^23^. Moreover, the peak wavelength of reflection encounters a red-shift of 10 nm because of the surface roughness (Fig. 6b). This can be explained by the incoherent effect caused by the local surface variation that disrupts partially the coherent scattering of a thin 1D photonic crystal i.e. the lamina. Hence, the local thickness variation of the lower lamella weakens the iridescent property of the scales towards UV spectral region.

**Figure 7:**
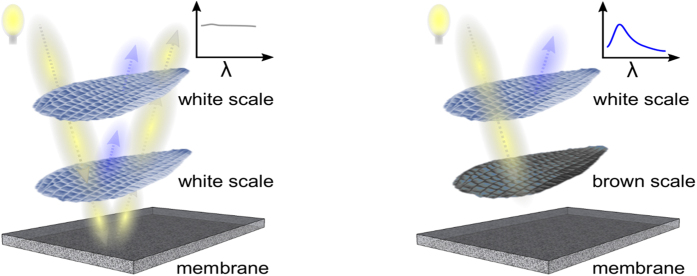
The mechanism of structural colouration by scale stacking in *Hypolimnas salmacis* butterfly wing. Schematics of scale stacking colouration mechanism of *Hypolimnas salmacis* butterfly wing demonstrating the role of a diffusive white wing membrane and an absorbing brown scale on colour appearance of a translucent white scale.

Micro-ridges in butterfly scales usually act as good scatters for longer wavelengths^36^. Again, windows created by cross-ribs are often reported to be responsible for the diffusion of the incoming or outgoing light^6^. This can explain the translucency of the white scales when white light passes through the upper lamina. Due to the high translucency of the white scales, the weak blue reflection from the lower thin lamina is eliminated by the underlying incoherent diffuse scattering of the wing membrane. The broadband absorbing pigment melanin in black scales effectively reduces stray-light and back-scattering, at the same time uncovers the blue appearance of the white scales. The overall mechanism of structural colouration by scale stacking in *Hypolimnas salmacis* wing is sketched in Fig. 7. The pigment melanin is also found in other blue, e.g. *Morpho* butterflies and mainly prevents the decrease in saturation of the colour^37^. The thin lower lamina in *Morpho* butterflies works as a thin reflector too, but the intense blue reflection of the scales is dominated by the upper lamina multi-layered lamellar architecture^38^. In contrast, the upper lamina of *Hypolimnas salmacis* white scales works as a broadband light scatterer.

The typical colours of butterfly wing scales may be solely produced from either micro- and nanoarchitectures, or pigments, but most frequently from a combination of both^37^,^39^. This is in fact also true for other animals in nature^40^,^41^,^42^. Often the cover scales of butterfly wings consist of complex architectures with ridges, lamella, microribs, crossribs with or without pigments and contribute strongly to the wing colouration. Otherwise, the cover scales are reported to be transparent and scatter the underlying ground scale reflection^26^. Only a few examples of scale stacking were reported so far and mainly described as an enhancer of scattering properties in terms of perception and brightness^23^,^43^. Spectral alteration by scale stacking was only recently observed in small regions of European *Nymphaline* butterflies^44^. A similar colouration mechanism is also found in the feathers of the bird *Steller’s Jay* where the colour difference of white and blue feathers arises primarily due to the inherent melanin pigment content difference below spongy nanostructures^45^,^46^.

In summary, we experimentally demonstrated that the blue wing regions of the *Hypolimnas salmacis* originate from scale stacking of white translucent scales on top of brown absorbing scales. Our single-scale micro-spectrometry and thin film simulation showed that the blue colouration is caused by the lower thin lamina of the white scales. Our detailed study also revealed the cause of the weak iridescence of *Hypolimnas salmacis* and might lead to better understanding of mating preferences and signal variation in other *Hypolimnas* butterflies^3^,^19^. Moreover, such a mechanism of colour interplay with different thin plates might be useful for technical applications in the field of nano-optics and photonics. For instance, by tuning the specular and/or diffuse reflection factor of a top optical layer, and the absorption properties of a bottom film, different optical signatures might be encoded.

## Methods

### Sample preparation and imaging

Dried samples of *Hypolimnas salmacis* were kindly supplied by the Stadtpark Mannheim GmbH, Germany and a dried sample was bought from Bug Under Glass©, USA. Scales were carefully removed from the wings with tweezers for subsequent imaging by optical microscopy. A stereo-microscope (SteREO Discovery.V8, Carl Zeiss Microscopy GmbH, Germany) was used in reflection mode to image the scales under epi-illumination condition (Fig. 1)

For the imaging by scanning electron microscopy (SEM), the examined regions of the butterfly wings were coated with a 15 nm thin gold layer (K575X sputter coater, Quorum Technologies Ltd.). Surface patterns were subsequently imaged by SEM (SUPRA^®^ 60 VP, Carl Zeiss Microscopy GmbH, Germany) operated at 5 kV (see Fig. 2a–d).

For cross-section imaging, small pieces of wings were embedded in epoxide resin. First, the pieces were dipped in a combination of 70% acetone and 30% epoxide mix (42.4 g Glycidether, 29.6 g DDSA, 18.4 g MNA, all chemicals from SERVA Electrophoresis GmbH, Germany). Subsequently, they were exposed to vacuum for a few minutes to remove air bubbles. Afterwards, the mixture was shaken for an hour. This step was repeated first with a combination of 30% acetone and 70% epoxide mix, then twice with 100% epoxide mix to make sure the viscous resin penetrated into all the tiny cavities of the butterfly scales. The resin-infiltrated wing pieces were placed into a silicone mold and covered with epoxide mix plus accelerator (10 g epoxide mix and 0.265 g BDMA) and baked at 65 °C for 2 days. The polymerized block was removed from the mold and trimmed to expose the wing piece. Thin (≈70 nm) sections from the block-face were prepared using an ultramicrotome (Leica Microsystems, Germany) and transferred to small pieces of silicon wafer for SEM imaging. The cross section shown in Fig. 2e) was imaged at 1.5 kV in a SEM (Ultra, Carl Zeiss Microscopy GmbH, Germany).

### Optical spectroscopy

The macroscopic reflection spectra of an intact *Hypolimnas salmacis* wing shown in Fig. 3 were recorded with a UV-Vis spectrometer (Lambda 1050, PerkinElmer Inc., USA). The total diffuse reflection was measured with an InGaAs 150 mm integrating sphere averaging over a 2 mm^2^ area on different regions (blue, white, brown) of an intact wing.

A customized Zeiss Axio microscope was used for the micro-spectroscopic analysis of individual scales (Fig. 4) with a spot size of ≈25 *μ*m in bright field (BF) reflection mode with a halogen lamp in Koehler illumination. Unpolarised light from the halogen lamp was illuminated via a 10X objective (EC Epiplan-APOCHROMAT, Zeiss) with a numerical aperture of NA = 0.3. The reflected light was collected with a spectrometer (AvaSpec-HS2048, Avantes, UK) through a 200 *μ*m core optical fiber (Avantes, UK) mounted in confocal configuration.

The angular-resolved specular reflection was measured using home-built optical goniometric setup (Fig. 6b). A light source from a stabilised Tungsten light source (SLS201, Thorlabs, USA) is collimated with a pinhole and a long working distance objective lens to form a 50 *μ*m wide parallel incident beam that illuminates a single scale at a fixed angle. The specularly reflected light is detected at different angles with an aperture of 2° and coupled into an optical fiber connected to the spectrometer (AvaSpec-ULS2048x64-USB2 Avantes, USA). All the spectra reported are referenced to a lambda/20 UV fused silica mirror (Thorlabs, USA).

## Additional Information

**How to cite this article**: Siddique, R. H. *et al*. Colour formation on the wings of the butterfly *Hypolimnas salmacis* by scale stacking. *Sci. Rep.* **6**, 36204; doi: 10.1038/srep36204 (2016).

**Publisher’s note:** Springer Nature remains neutral with regard to jurisdictional claims in published maps and institutional affiliations.
